# Supplementary material for: First Report of the Yezo Virus Isolates Detection in Russia
Source: Viruses. 2025 Aug 15;17(8):1125. doi: 10.3390/v17081125 (PMC12390643; doi:10.3390/v17081125)
Supplement: Supplementary file 1 [file viruses-17-01125-s001.zip › viruses-3745335-supplementary.pdf]

Table S1. Geographic coordinates of tick collection. Geographic coordinates of YEZV positive ticks are highlighted in bold.

Russia region	Tick species	No. ticks examined	Description of the tick collection site	Latitude (°)	Longitude (°)
Khabarovsk Territory	*I. persulcatus*	79	**Bikinsky municipal district***	**47,248631**	**134,395772**
			Bikinsky municipal district	47,248713	134,395811
			Bikinsky municipal district	47,248845	134,395932
Primorsky Territory	*I. persulcatus*	368	Valley of the river Dukhovskoy	44,083143	131,423390
			Valley of the river Dukhovskoy	44,170417	131,689235
			Valley of the river Dukhovskoy	44,256722	131,661074
			Valley of the river Dukhovskoy	44,428951	131,379466
			Valley of the river Dukhovskoy	44,329703	131,539691
			Valley of the river Dukhovskoy	44,505874	131,532894
			Valley of the river Dukhovskoy	44,638691	131,767891
			Valley of the river Dukhovskoy	44,720167	131,672727
			Valley of the river Dukhovskoy	44,570245	131,562026
			Valley of the river Dukhovskoy	44,635241	131,551344
			Valley of the river Talovka	45,681952	134,098788
			Valley of the river Talovka	45,554263	134,176474
			**Valley of the river Talovka***	**45,498478**	**134,243477**
			Valley of the river Talovka	45,640553	133,818151
			Valley of the river Talovka	45,342207	134,044118
			Valley of the river Talovka	45,402239	134,083382
			Valley of the river Talovka	45,482140	134,043438
			Valley of the river Talovka	45,573299	134,206577
			Valley of the river Talovka	45,644627	133,823978
			Valley of the river Talovka	45,751786	134,102673
			Valley of the river Talovka	45,372823	133,698046
			Valley of the river Talovka	45,527931	133,751605
Transbaikal Territory	*I. persulcatus*	150	Duldurga district, Alkhanai National Park	50,896344	113,227117
			Duldurga district, Alkhanai National Park	50,908695	113,226269

			**Duldurga district, Alkhanai National Park***	**50,924285**	**113,282046**
			Duldurga district, Alkhanai National Park	50,903155	113,186729
			Duldurga district, Alkhanai National Park	50,907904	113,170031
			Duldurga district, Alkhanai National Park	50,883139	113,288686
			Duldurga district, Alkhanai National Park	50,924649	113,224595
			Duldurga district, Alkhanai National Park	50,904690	113,249654
			Duldurga district, Alkhanai National Park	50,885218	113,221662
			Duldurga district, Alkhanai National Park	50,891046	113,250821
			Duldurga district, Alkhanai National Park	50,899058	113,196256
Altai Republic	*I. persulcatus*	79	Gorno-Altaisk, "Health Path"	51,975826	85,866044
			Gorno-Altaisk, "Health Path"	51,981807	85,907799
			Gorno-Altaisk, "Health Path"	51,954287	85,892263
			Gorno-Altaisk, "Health Path"	51,901292	85,914597
			Gorno-Altaisk, "Health Path"	51,971638	86,019472
Arkhangelsk region	*I. persulcatus*	428	Onega district, Onega city, Onega mountain surroundings	63,931518	38,126455
			Kholmogory district, village Usolye	63,624994	41,626787
			Onega district, village Pokrovskaya	64,018865	38,104973
			Ustyansky district, road near the cemetery of the village of Tarasonavolodskaya	61,105801	43,248822
			Ustyansky district, Oktyabrsky settlement	61,093697	43,167128
			Shenkursky district, village V. Gora	61,766265	42,564949
			Shenkursky district, village Baranovskaya	61,731872	42,535366
			Shenkursky district, village Tyrlinskaya	61,627156	42,464067
			Onega district, village Tamitsa	64,165019	38,036261
			Konosha district, village Voloshka	61,332125	40,086724
			Konoshsky district, village Papinskaya, forest road	60,988866	41,133781

			Konoshsky district, village Zaruchevskaya	60,904492	40,876803
			Konosha district, surroundings of Solginsky settlement	61,054088	41,331572
			Velsky district, Velsk	61,072196	42,123450
			Velsky district, village Shilovskaya	61,018577	42,041606
			Velsky district, village Lenina-Ulyanovskaya	61,072772	42,013390
			Shenkursky district, village Ust-Padenga	61,913484	42,645459
			Shenkursky district, village Spasskoe	62,074265	42,876119
			Velsky district, village Dolmatovo	61,541466	42,348880
			Velsky district, village Pokrovskaya	61,095114	42,180411
Bryansk region	*I. ricinus*	51	Trubchevsky district, village Ratchino	52,658471	33,829544
			Vygonichsky district, village Paluzh'e	53,160798	34,202621
			Bryansk region, Megapolis-Park	53,267439	34,287274
			Brasovsky district, village Klinskoe	52,707421	34,624654
			Bryansk district, village Steklyannaya Raditsa	53,380504	34,483100
			Bryansk region, settlement Belye Berega, river Snezhet	53,222760	34,646957
			Vygonichsky district, 41 km of the A-240 highway	53,232936	34,323348
			Mglinsky district, village Polkhovka	53,117750	33,083765
			Mglinsky district, southwestern outskirts of the Kabanovka farm	53,123848	32,818869
			Dubrovsky district, village Aleshnya	53,639172	33,439668
			Vygonichsky district, Nikolsky village, Revna river	52,973528	34,068862
			Bryansk, forest park «Solov'i»	53,272279	34,355765
			Bryansk, Maltsevskaya station	53,282329	34,384188
			Bryansk district, village Koltovo	53,276384	34,124683

			Dubrovsky district, village Bolshaya Ostrovnya	53,743659	33,289981
			Navlinsky district, village Navlya	52,832488	34,465331
			Bryansk region, village Orlovsky Dvoriki	53,395797	34,444876
			Bryansk district, village Dobrun	53,201028	34,444876
Irkutsk region	*I. persulcatus*	200	Irkutsk region, Baikal tract	52,086177	104,587848
			Irkutsk region, Baikal tract	52,082363	104,586404
			Irkutsk region, Baikal tract	52,071273	104,594777
			Irkutsk region, Baikal tract	52,075266	104,584672
			Irkutsk region, Baikal tract	52,064707	104,606613
			Irkutsk region, Baikal tract	52,073935	104,618306
			Irkutsk region, Baikal tract	52,073625	104,593489
			Irkutsk region, Baikal tract	52,070582	104,587620
			Irkutsk region, Baikal tract	52,056276	104,620904
			Irkutsk region, Baikal tract	52,051838	104,621626
			Irkutsk region, Baikal tract	52,032127	104,645443
			Irkutsk region, Baikal tract	52,026887	104,642845
			Irkutsk region, Baikal tract	52,057962	104,664642
			Irkutsk region, Baikal tract	52,026976	104,663920
			Irkutsk region, Baikal tract	52,062222	104,635772
			Irkutsk region, Baikal tract	52,028308	104,647897
			Irkutsk region, Baikal tract	52,049885	104,655692
			Irkutsk region, Baikal tract	52,045091	104,643134
			Irkutsk region, Baikal tract	52,057341	104,620327
			Irkutsk region, Baikal tract	52,024489	104,645588
			Irkutsk region, Baikal tract	52,013918	104,663776
			Irkutsk region, Baikal tract	52,011519	104,676623
			Irkutsk region, Baikal tract	52,015162	104,681242
Ivanovo region	*I. persulcatus*	135	Lezhnevsky district, village Lezhnevskoye	56,805139	40,873389
			Ivanovo district, village Novotalitskoye	57,018139	40,835556

			Shuisky district, village Kitovskoye	56,860167	41,232944
			Rodnikovsky district, village Kaminskoe	57,093500	41,424417
Kaliningrad region	*I. ricinus*	860	Baltiysk city	54,645485	19,883146
			Zverevo, "Bolshakovsky" forest	54,329530	21,426641
			Dimitrievka	54,419356	22,459863
			Kamarici, forest near Lake Gołdap	54,358521	22,310539
			Kashtanovka	54,869142	20,494657
			Sosnovka, forest on the bank of the river "Krasnaya"	54,386495	22,431705
			Malinovka	54,827703	20,527954
			Mamonovo	54,444969	19,907112
			Pugachevo, "Neman" forest	55,039780	22,559013
			Sosnovka	54,939628	20,509282
			Veselovka	54,720645	20,286891
			Zelenogradsk, the coast of the Zelenogradka river	54,938118	20,505266
			Ilyichevka, "Zvonkiy" forest	54,486761	20,179260
Kemerovo region	*I. persulcatus*	289	Yashkinsky municipal district, village Pisanaya	55,308444	86,159361
			Kemerovo municipal district, village Podyakovo	55,571806	85,824028
			Kemerovo, st. 62nd passage	55,663263	85,625617
Khakassia Republic	*I. persulcatus*	150	Beysky district, Tabat river	52,962593	90,713611
			Beysky district, Tabat river	52,931459	90,745539
			Beysky district, Tabat river	52,925664	90,662455
			Beysky district, Tabat river	52,899173	90,740849
			Beysky district, Tabat river	52,892785	90,681618
			Beysky district, Tabat river	52,877662	90,869416
			Sayanogorsk, dacha community «Bolshoy Karak»	53,056745	91,409172

			Sayanogorsk, dacha community «Bolshoy Karak»	53,082324	91,487449
			Sayanogorsk, dacha community «Bolshoy Karak»	53,059221	91,277336
			Sayanogorsk, dacha community «Bolshoy Karak»	53,017108	91,459984
			Sayanogorsk, dacha community «Bolshoy Karak»	53,027021	91,501182
			Sayanogorsk, dacha community «Bolshoy Karak»	53,076549	91,370720
Kirov region	*I. persulcatus*	85	Kirov, Poroshino	58,604311	49,802562
			Kilmez district, village Osinovka	57,414221	50,843090
			Kirov, village Bakhta	58,609085	49,408835
			Slobodskoy district, village Kiseli	58,661377	49,789406
			Kirovo-Chepetsk district, village Perekop	58,496228	49,919310
			Bogorodsky district, village Taranki	57,966269	50,574267
Komi Republic	*I. persulcatus*	82	Knyazhpogotsky district, village Lyali, side of a dirt road	62,268110	50,675310
			Priluzsky district, village Porubkepovskaya, forest road	60,462321	48,592341
			Koygorodsky district, Koygorodok outskirts, forest road	60,464457	50,967432
			Syktyvdinsky district, peat bogs, side of a dirt road	61,550500	50,657332
			Sysolsky district, village Pyeldino	61,005646	50,154578
			Priluzsky district, village Ob'yachevo	61,352613	49,617241
Krasnoyarsk Territory	*I. persulcatus*	200	Lake «Linevo», stationary	55,409938	89,087021
			Neighborhood of Ust-Parnaya village	55,411612	89,125355
			Surroundings of Sharypovo	55,548300	89,184555
			Lake «Linevo», stationary	55,409938	89,087020
			Surroundings of the village Kholmogorskoe	55,468730	89,153362

Mari El Republic	*I. persulcatus*	55	Volozhsk, Russkaya Lugovaya district	55,895929	48,292738
			Yoshkar-Ola, «Sosnovaya Roshcha» forest park	56,617831	47,930911
			Medvedevsky district, garden association «Avtodorozhnik»	56,656148	47,325280
			Kuzhenersky district, village Pamashnur	56,773353	49,008557
Novosibirsk region	*I. persulcatus*	107	Toguchinsky district, village Lekarstvennoe	55,002237	83,647795
			Iskitimsky district, village Talmenka	54,709513	83,278689
			Iskitimsky district, village Burmistrovo	54,636548	82,835624
			Novosibirsk district, village Berezovsky	54,941806	83,287833
			Novosibirsk region, village Baryshevo	54,852722	83,206500
			Suzunsky district, village Verkh-Suzun	53,673500	82,335500
			Suzunsky district, village Malyshevo	53,751889	82,074139
Smolensk region	*I. ricinus*	285	Smolensk, Krasny Bor settlement	54,791820	31,921566
			Smolensk district, village Borovaya	54,772367	31,898949
			Smolensk district, village Borovaya	54,770437	31,903885
			Krasninsky district, village Mankovo	54,599623	31,489993
			Demidovsky district, village Zabor'e	55,394070	31,565717
			Demidovsky district, village Zabor'e	55,406254	31,564515
			Yershichsky district, village Poselki	53,743173	32,541894
			Yershichsky district, village Poselki	53,743173	32,541894
			Roslavl district, village Koski	54,043366	33,022601
			Roslavl district, village Koski	54,030462	33,040454
			Shumyachsky district, village Gorolets	53,851102	32,333346
			Shumyachsky district, village Gorolets	53,845430	32,332646
Tomsk region	*I. persulcatus*	340	Tomsk, «Burevestnik» stadium	56,446808	84,977540
			Tomsk, Kolarovsky tract	56,450820	84,980653
			Tomsk, st. Continental	56,443435	85,005635
			Tomsk, coastal zone of the Tom River	56,447852	84,968828
			Tomsk, Botanical Garden	56,452972	85,001570

			Tomsk, coastal zone of the Tom River	56,449915	84,957112
			Tomsk, ski resort	56,468051	85,033955
			Tomsk, Anikino microdistrict	56,406504	84,989166
			Tomsk, village Basandaika	56,400464	84,984288
			Tomsk, Akademgorodok	56,475670	85,041239
			Tomsk region, village Bogashevo	56,375149	85,100275
			Tomsk region, village Mezheninovskoye	56,359071	85,350902
			Tomsk, "Sunny Grove"	56,515370	85,056172
			Tomsk region, village Zorkaltsevskoe	56,480735	84,872351
Tver region	*I. persulcatus*	170	Kalininsky district, village Novinki	56,876164	35,539768
			Rzhevsky district, «Nizhny Bor» settlement	56,241760	34,366784
			Torzhok district, village Mitino	57,105184	34,979375
			Konakovsky district, village Vakhromeevo	56,675460	36,702248
Tyumen region	*I. persulcatus*	137	Ishimsky district, village Klepikovskoye	56,012889	69,443333
			Sladkovsky district, village Stepnovskoe	55,608500	70,188222
			Berdyuzhsky district, village Okunevskoye	55,785611	68,688333
			Armizonsky district, village Krasnoorlovskoye	55,968806	67,942139
			Aromashevsky district, village Maloskaredinskoye	56,800369	69,221559
Tyva Republic	*I. persulcatus*	210	Kaa-Khem district, Koptu	51,593495	95,352015
			Kaa-Khem district, Koptu	51,573868	95,365404
			Kaa-Khem district, Koptu	51,570241	95,365748
			Kaa-Khem district, Koptu	51,564478	95,365061
			Todzhinsky district, Tozhu pass	52,435407	96,488497
			Todzhinsky district, Tozhu pass	52,429424	96,517624
			Todzhinsky district, Tozhu pass	52,435896	96,517216
			Todzhinsky district, Tozhu pass	52,421208	96,537222

			Todzhinsky district, Tozhu pass	52,432660	96,539671
			Todzhinsky district, Tozhu pass	52,439878	96,537629
			Todzhinsky district, Tozhu pass	52,447096	96,507009
			Todzhinsky district, Tozhu pass	52,442367	96,453932
			Todzhinsky district, Tozhu pass	52,433158	96,420453
			Todzhinsky district, Tozhu pass	52,425689	96,422903
			Todzhinsky district, Tozhu pass	52,435708	96,481386
			Todzhinsky district, Tozhu pass	52,431836	96,511411
			Todzhinsky district, Tozhu pass	52,436412	96,412098
			Todzhinsky district, Tozhu pass	52,426203	96,417295
			Todzhinsky district, Tozhu pass	52,392037	96,360709
			Todzhinsky district, Tozhu pass	52,381113	96,376299
			Todzhinsky district, Tozhu pass	52,433596	96,507371
			Todzhinsky district, Tozhu pass	52,418456	96,449052
			Todzhinsky district, Tozhu pass	52,409299	96,455403
			Todzhinsky district, Tozhu pass	52,424090	96,304125
			Todzhinsky district, Tozhu pass	52,432539	96,279297
			Todzhinsky district, Tozhu pass	52,420217	96,265439
			Tandinsky district, Durgen Canyon	51,049363	94,540974
			Tandinsky district, Durgen Canyon	51,048481	94,544240
			Tandinsky district, Durgen Canyon	51,045063	94,535258
			Tandinsky district, Durgen Canyon	51,043019	94,532888
			Tandinsky district, Durgen Canyon	51,040615	94,538046
			Tandinsky district, Durgen Canyon	51,038020	94,530551
Udmurt Republic	*I. persulcatus*	600	Mozhginsky district, forest of the Muslim cemetery	56,434933	52,299947
			Votkinsk district, camp «Chaika»	57,102109	53,941665
			Izhevsk, Yakshur-Bodinsky tract	56,928214	53,125277
			Sarapul, Northern district	56,464831	53,749415
			Sarapul, village Kotovo	56,189102	53,720358

			Sarapul, Old Izhevsk Highway	56,486061	53,738211
			Sarapul district, village Kostino	56,390617	53,753049
			Sarapul district, village Yaromaska	56,520904	53,777178
			Glazovsky district, village Simashur	58,193896	52,739647
Vologda region	*I. persulcatus*	258	Vytegorsky district, village Ozerki	60,961599	36,604140
			Vashkinsky district, village Vasilyevskaya	60,280138	38,019220
			Kirillovsky district, village Topornya	59,765785	38,389472
			Velikoustyugsky district, Mardensky territorial entity, village Krasnoe pole	60,783251	46,237931
			Kichmengsko-Gorodetsky district, village Zvezda	59,837943	45,611773
			Totemsky district, village Medvedevo	60,029842	43,028162
			Tarnogsky district, village Tyupriha	60,440195	43,284324
			Babushkinsky district, village Ledenga	59,926351	42,889789
			Cherepovets city	59,166239	37,879808
			Ustyuzhensky district, municipal district "Zalesskoye"	58,737561	36,221333
			Chagodoshchensky district, Megrinskoye rural settlement, village Megrino	59,149294	35,591154
			Mezhdurechensky district, village Karpovskoye	59,164933	40,496273
			Vologda district, "Avtomobilist-1"	59,047911	40,085570

*- Tick collection sites where YEZV positive ticks were found

Table S2. Oligonucleotide primers used for targeted library enrichment on NGS.

Oligonucleotide name	Oligonucleotide sequence	Coordinate
YV_L_1F	ACATCTATCCTGCAATCCC	F1
YV_L_1R	GGTGYCTYCCAACTTTCCC	R962
YV_L_2F	CTACTGTCCAAGCCAACCA	F869
YV_L_2R	TCAAAMAGAACCTCGCACTC	R2099
YV_L_3F	AGTGCCCATAGCAATYATC	F2017
YV_L_3R	CTGGCTATACCTTTGATTGCAT	R3272
YV_L_4F	TCATGGTTGCCAACATCAGC	F2874
YV_L_4R	GTCAGACTTGTCCACTATA	R3976
YV_L_5F	AGCCTGCAAGATAATGAACAC	F3751
YV_L_5R	ATACCAGCTGTAAGTAAAGCC	R4836
YV_L_6F	GACAGGCAACTTTCTTGACC	F4597
YV_L_6R	TTTCTGTGCCTCTGTTAGTGT	R5786
YV_L_7F	TCATTCAAGCYTTCACGGAG	F5493
YV_L_7R	CTCATCAGGCTTCATTGCAT	R6684
YV_L_8F	GCAWTGAAACGGCTTACTTC	F6500
YV_L_8R	TCTTACTTATTGCTCCGCTGA	R7764
YV_L_9F	TGGCAAGCAAATACAAACTCG	F7446
YV_L_9R	ATCTAACAAGGCCAAATGTCC	R8661
YV_L_10F	AGGATTATGACGGATTCCCT	F8559
YV_L_10R	GGCCTCCGTAATCAACCCTG	R9577
YV_L_11F	ATGCAGTTTTCAGAGCCGAA	F9377
YV_L_11R	GCGGTTCACTACAGTCACA	R10622
YV_L_12F	CCTGCTTCCAACCTATCCAA	F10450
YV_L_12R	AGACCTCTGTCATCTAGGCTR	R11371
YV_L_13F	GAGCTAAGCAGCAAGTCACA	F10901
YV_L_13R	GTTCCTGCATACCCCCCTATTA	R12101
YV_M_1F	ACTTGCAGCGACCCCCCCAAAG	F1
YV_M_1R	TCCACCACTGCCTTACAGCC	R851
YV_M_2F	ATCAGARAATATAAAGGTCAGC	F763
YV_M_2R	GCTCGACAGACATAGATTGC	R1631
YV_M_3F	CTGCTCCTAAAGGAAGTCTWG	F1518
YV_M_3R	CCCTATTTTGACTTTGCCAAC	R2397
YV_M_4F	GYCCAATTCAAGAATCRCCTA	F2217
YV_M_4R	ATCTACTAAGCCATTGAGGTTG	R3094
YV_M_5F	ATCTCTAGGAGGCATATCAGT	F2893
YV_M_5R	CTCYGACTCTTTTGGTGCTT	R3699
YV_M_6F	AACATTRGACAGTCCYGACCGG	F3535
YV_M_6R	AGTGGCAGCATACCCCCCTT	R4237
YV_S_1F	CGTGCTGCGACCCCCCAATAG	F1
YV_S_1R	TCCATCTTCTGCTGGTTGT	R840
YV_S_2F	GCAGGAACAAGAAGCACTC	F607
YV_S_2R	AGCCTTCTTTGATCTTRACA	R1273
YV_S_3F	CYAACATGTGTGCAACRGGA	F1152
YV_S_3R	CCTAGAAGCGGGAAGATGTTGT	R1676

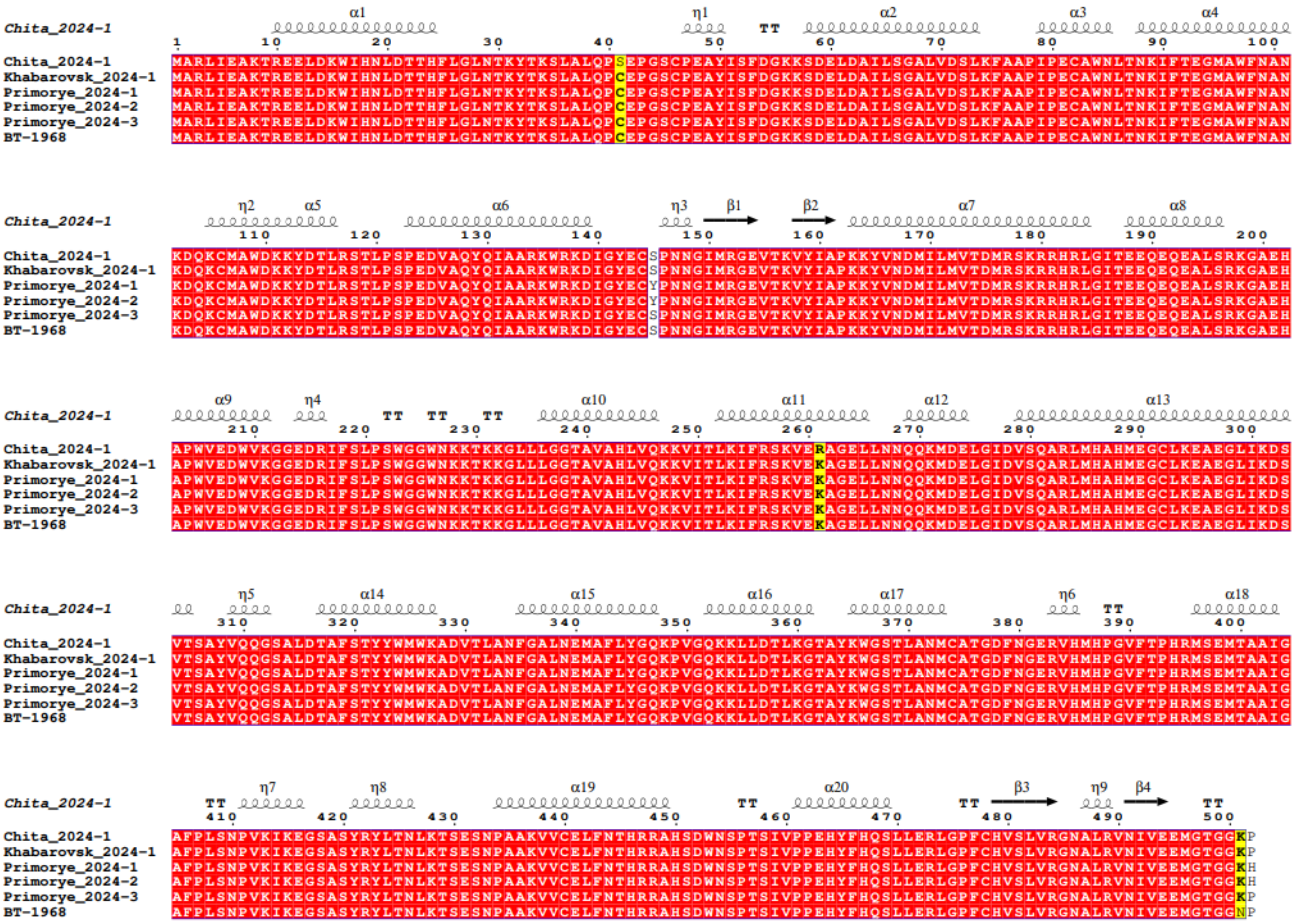

Figure S1. Sequence alignment of the Russian YEZV N isolates and YEZV N BT-1968 isolate (GenBank ID: LC790676, *I. persulcatus*, Japan): red boxes—100% aligned a.a. residues; yellow boxes—80% aligned a.a. residues; white boxes - unaligned.

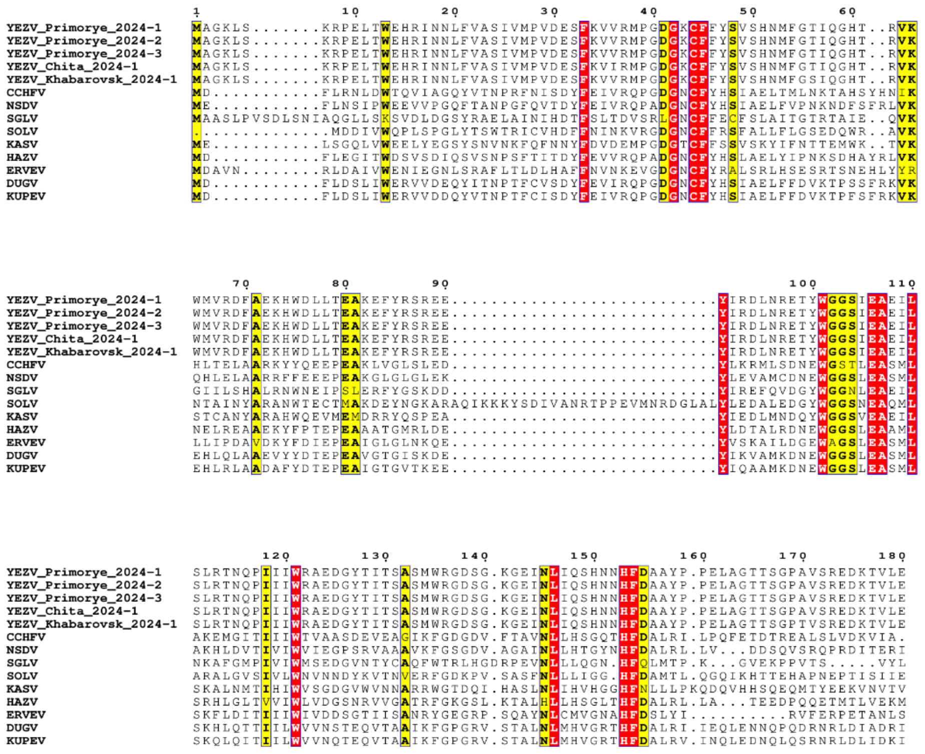

Figure S2. Sequence alignment of the Russian YEZV N isolates OTU-like domain of L-protein and other orthonairoviruses OTU-like domain of L-protein: red boxes—100% aligned a.a. residues; yellow boxes—80% aligned a.a. residues; white boxes - unaligned. The selected orthonairoviruses: CCHFV – *Orthonairovirus haemorrhagiae* (GenBank ID: AQX83296.1), NSDV – *Orthonairovirus nairobiense* (GenBank ID: XCB13529.1), SGLV – *Orthonairovirus songlingense* (GenBank ID: UXX19114.1), SOLV – Orthonairovirus soldadoense (GenBank ID: YP_010839838.1), KASV – *Orthonairovirus kasokeroense* (QNS29866.1), HAZV – *Orthonairovirus hazaraense* (GenBank ID: YP_009507850.1), ERVEV – *Orthonairovirus erveense* (GenBank ID: AFH89032.1), DUGV – *Orthonairovirus dugbeense* (GenBank ID: AAB18834.1), KUPEV – *Orthonairovirus amblyommae* (GenBank ID: ABY82502.1).

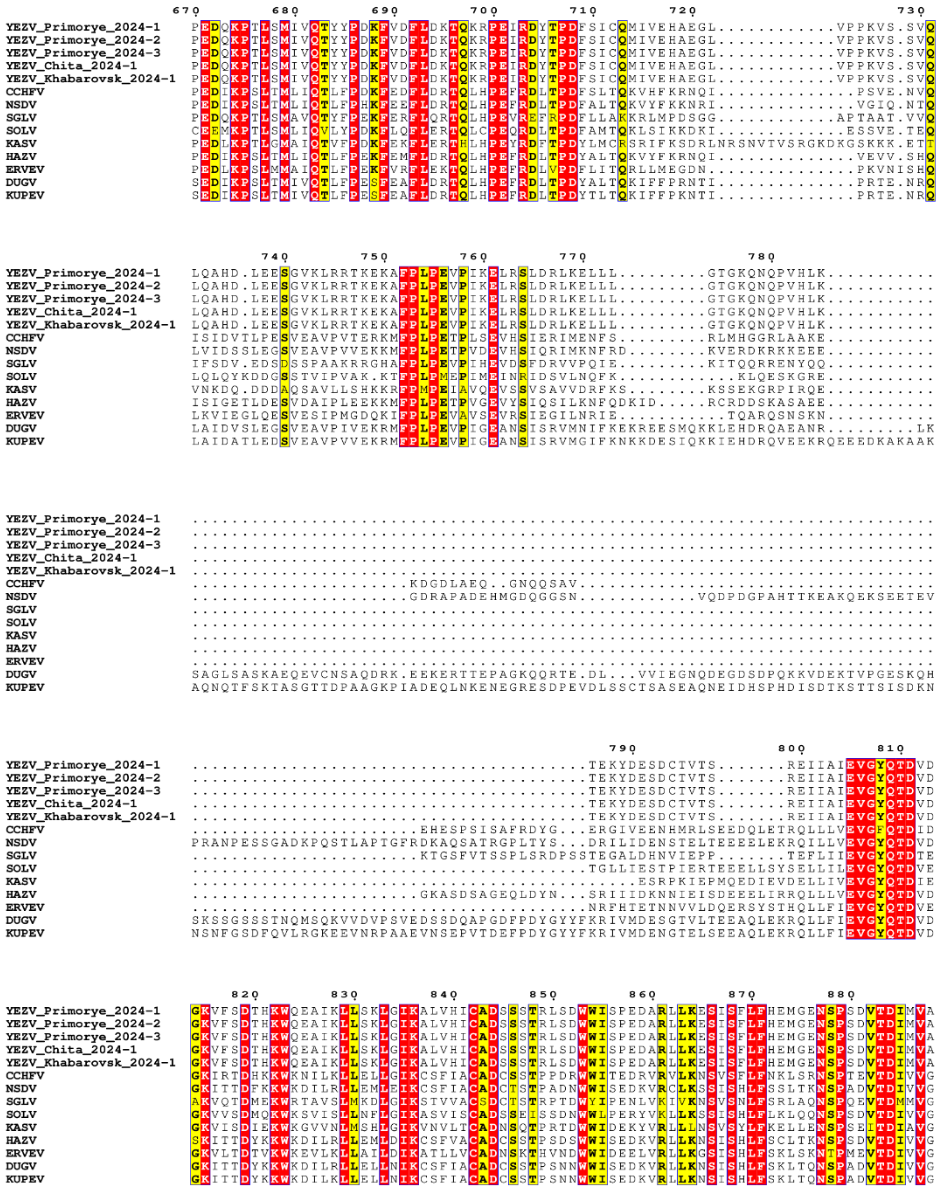

Figure S3. Sequence alignment of the Russian YEZV N isolates endonuclease domain of L-protein and other orthonairoviruses endonuclease domain of L-protein: red boxes—100% aligned a.a. residues; yellow boxes—80% aligned a.a. residues; white boxes - unaligned. The selected orthonairoviruses: CCHFV – *Orthonairovirus haemorrhagiae* (GenBank ID: AQX83296.1), NSDV – *Orthonairovirus nairobiense* (GenBank ID: XCB13529.1), SGLV – *Orthonairovirus songlingense* (GenBank ID: UXX19114.1), SOLV – *Orthonairovirus soldadoense* (GenBank ID: YP_010839838.1), KASV – *Orthonairovirus kasokeroense* (GenBank ID: QNS29866.1), HAZV – *Orthonairovirus hazaraense* (GenBank ID: YP_009507850.1), ERVEV – *Orthonairovirus erveense* (GenBank ID: AFH89032.1), DUGV – *Orthonairovirus dugbeense* (GenBank ID: AAB18834.1), KUPEV – *Orthonairovirus amblyommae* (GenBank ID: ABY82502.1).

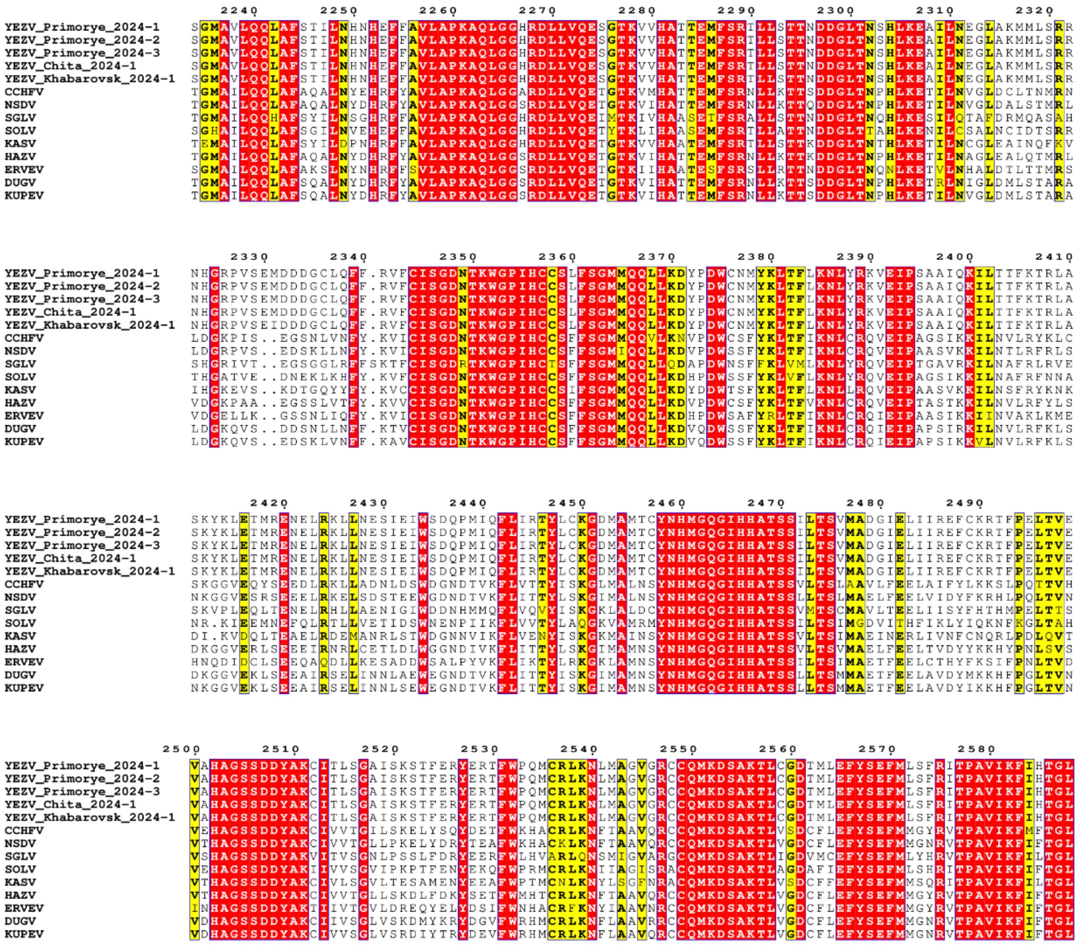

Figure S4. Sequence alignment of the Russian YEZV N isolates RdRp domain of L-protein and other orthonairoviruses RdRp domain of L-protein: red boxes—100% aligned a.a. residues; yellow boxes—80% aligned a.a. residues; white boxes - unaligned. The selected orthonairoviruses: CCHFV – *Orthonairovirus haemorrhagiae* (GenBank ID: AQX83296.1), NSDV – *Orthonairovirus nairobiense* (GenBank ID: XCB13529.1), SGLV – *Orthonairovirus songlingense* (GenBank ID: UXX19114.1), SOLV – *Orthonairovirus soldadoense* (GenBank ID: YP_010839838.1), KASV – *Orthonairovirus kasokeroense* (GenBank ID: QNS29866.1), HAZV – *Orthonairovirus hazaraense* (GenBank ID: YP_009507850.1), ERVEV – *Orthonairovirus erveense* (GenBank ID: AFH89032.1), DUGV – *Orthonairovirus dugbeense* (GenBank ID: AAB18834.1), KUPEV – *Orthonairovirus amblyommae* (GenBank ID: ABY82502.1).

Table S3. Comparison of amino acid sequences and tertiary structure of YEZV OTU-like domain with OTU-like domain of *Nairoviridae* family viruses.

PDB ID	Name of virus	TM-score	RMSD, Å	Aligned residues, a.a.	Amino acid sequence identity, %
7JMS	Hazara virus	0.91	1.87	150	28
6OAR	Kupe virus	0.9	1.9	148	30
3PHW	Crimean Congo Hemorrhagic Fever Virus	0.88	1.75	145	26
7Y5M	Tacheng Tick Virus 1	0.88	2.06	140	31
4HXD	Dugbe virus	0.91	1.78	147	30

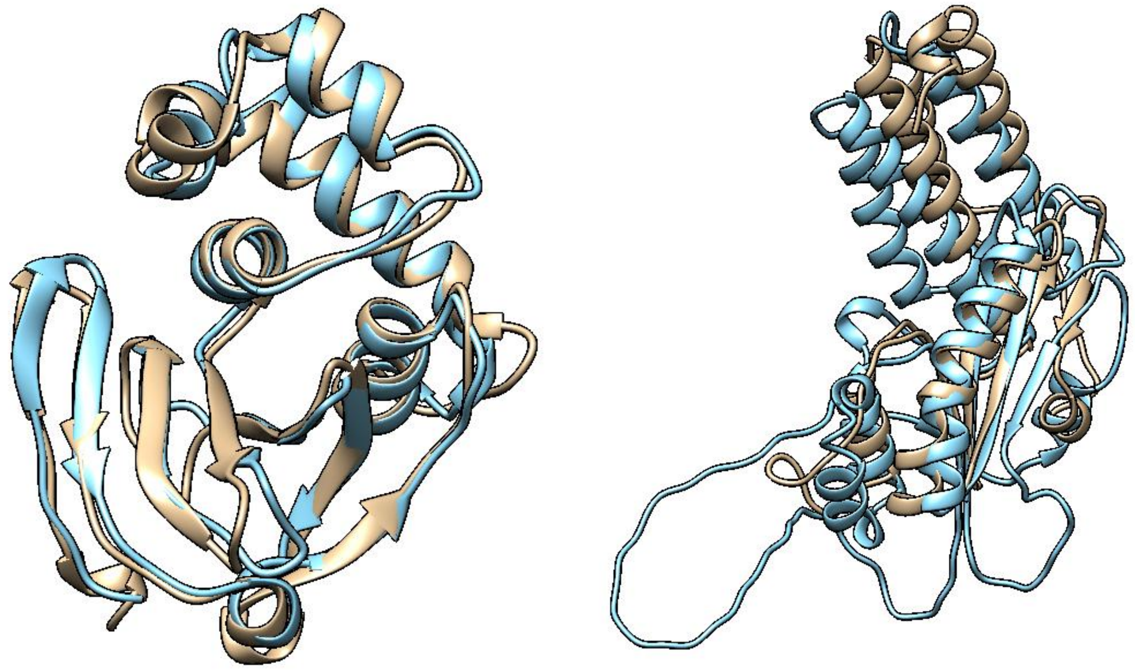

Figure S5. Imposition models of YEZV Primorye 2024-1 isolate proteins tertiary structures. (**left**) Imposition models of YEZV Primorye 2024-1 isolate OTU-like domain tertiary structure (blue) with *Orthonairovirus haemorrhagiae* OTU-like domain (PDB ID: 3PRP) (ivory); (**right**) Imposition models of YEZV Primorye 2024-1 isolate endonuclease domain tertiary structure (blue) with *Orthobunyavirus encephalitidis* endonuclease domain (PDB ID: 2XI7) (ivory).

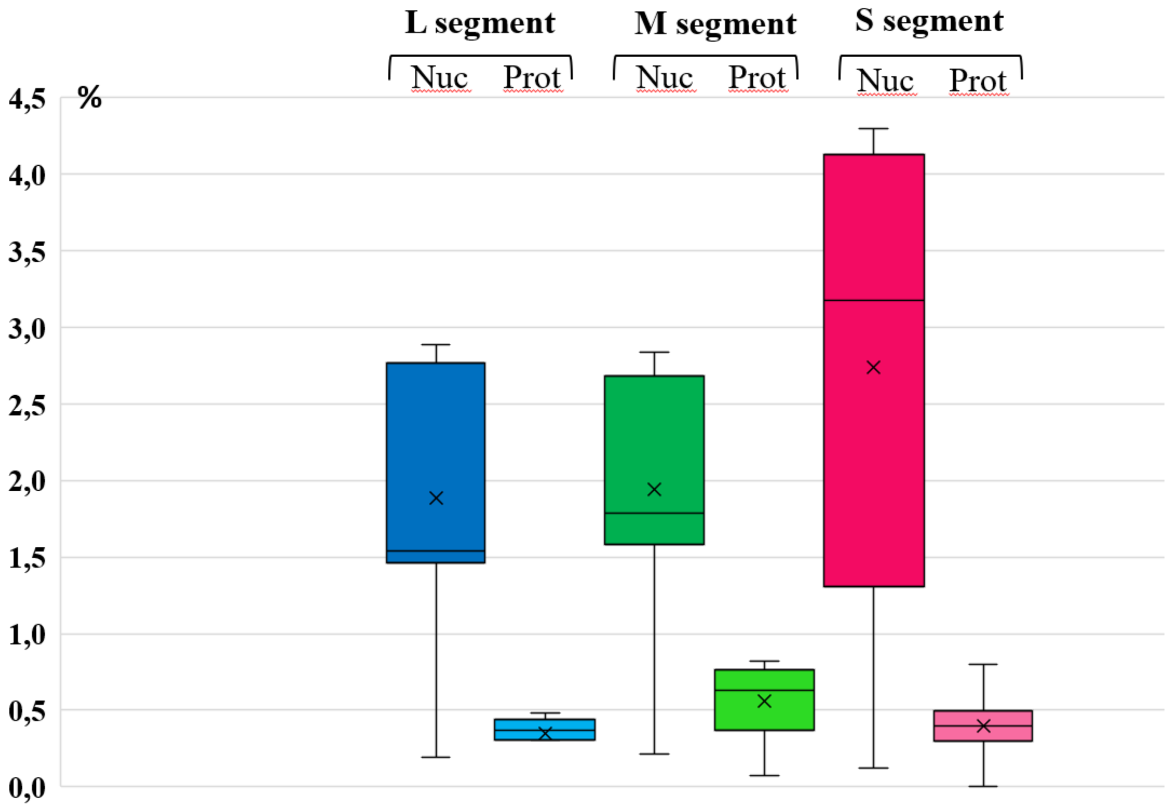

Figure S6. Level of sequence differences in identified Russian YEZV isolates.

Table S4. Identity level of nucleotide and amino acid sequences (%) of identified Russian YEZV isolates with a YEZV prototype isolates.

Name of YEZV isolate	Geographical name of location, host	Name of Russian YEZV isolate									
		Primorye 2024-1		Primorye 2024-2		Primorye 2024-3		Chita 2024-1		Khabarovsk 2024-1	
		nuc.	prot.	nuc.	prot.	nuc.	prot.	nuc.	prot.	nuc.	prot.
L segment											
TIGMIC 1	China: Heilongjiang, *I.persulcatus*	98,35	99,64	98,32	99,67	98,97	99,82	97,10	99,59	98,34	99,67
THQC1707B	China: Tonghua, *I.persulcatus*	95,39	99,34	95,37	99,31	95,45	99,36	95,19	99,39	95,53	99,26
YBQC1712	China: Yanbian, *I.persulcatus*	97,51	99,72	97,53	99,75	97,45	99,75	97,39	99,77	97,57	99,69
MDJ014	China: Heilongjiang, *Homo Sapiens*	98,56	99,72	98,52	99,75	98,59	99,75	97,33	99,67	98,56	99,75
MDJ486	China: Jilin, *Homo Sapiens*	98,32	99,52	98,29	99,54	99,02	99,94	97,03	99,41	95,28	99,53
BT-2155	Japan, *I.persulcatus*	98,41	99,54	98,37	99,57	98,82	99,72	97,15	99,49	98,41	99,57
HH011-2020	Japan, *Homo Sapiens*	97,42	99,62	97,44	99,64	97,38	99,65	97,36	99,67	97,51	99,59
M segment											
TIGMIC 1	China: Heilongjiang, *I.persulcatus*	98,12	99,70	98,12	99,63	98,60	99,93	97,72	99,41	98,46	99,70
THQC1707B	China: Tonghua, *I.persulcatus*	96,41	98,82	96,39	98,75	96,08	98,89	96,34	98,89	96,11	98,67
YBQC1712	China: Yanbian, *I.persulcatus*	97,62	99,41	97,65	99,34	97,48	99,48	95,35	99,26	97,36	99,26
MDJ014	China: Heilongjiang, *Homo Sapiens*	98,69	99,78	98,72	99,70	98,43	99,85	98,05	99,48	98,29	99,63
MDJ486	China: Jilin, *Homo Sapiens*	98,27	99,54	98,34	99,48	98,97	99,78	97,81	99,26	98,55	99,56
BT-2155	Japan, *I.persulcatus*	97,89	99,56	98,43	99,98	98,74	99,78	97,86	99,26	98,60	99,56
HH011-2020	Japan, *Homo Sapiens*	97,46	99,50	98,48	99,48	97,36	99,63	97,65	99,41	97,27	99,48
S segment											
TIGMIC 1	China: Heilongjiang, *I.persulcatus*	95,51	99,40	95,64	99,40	95,69	99,80	98,71	99,40	98,50	99,80
THQC1707B	China: Tonghua, *I.persulcatus*	95,51	99,40	95,63	99,40	98,55	99,81	98,23	99,40	98,92	99,81
YBQC1712	China: Yanbian, *I.persulcatus*	95,50	99,60	95,64	99,68	98,55	100	98,42	99,40	98,92	100
MDJ014	China: Heilongjiang, *Homo Sapiens*	95,45	99,40	95,57	99,40	98,48	99,80	98,23	99,40	98,56	99,80
MDJ486	China: Jilin, *Homo Sapiens*	95,32	99,40	95,45	99,40	98,23	99,80	98,29	99,40	98,67	99,80
BT-2155	Japan, *I.persulcatus*	95,35	99,40	95,57	99,40	98,42	99,80	98,57	99,40	98,80	99,60
HH011-2020	Japan, *Homo Sapiens*	94,82	99,41	94,82	99,40	93,43	99,40	93,30	99,00	93,36	99,40

Table S5. Comparison of tertiary structure models of different YEZV N isolates.

Name of YEZV isolate (pLDDT)		TM-score	RMSD, Å
Chita 2024-1 (87.4)	Primorye 2024-1 (86.8)	0.95	2,01
	Khabarovsk 2024-1 (86.8)	0.94	2,34
	Primorye 2024-2 (86.9)	0.92	2.68
	Primorye 2024-3 (87.0)	0.98	1.12
Khabarovsk 2024-1 (86.8)	Primorye 2024-1 (86.8)	0.99	0.87
	Primorye 2024-2 (86.9)	0.97	1.39
	Primorye 2024-3 (87.0)	0.97	1.67
Primorye 2024-1 (86.8)	Primorye 2024-2 (86.9)	0.98	1.22
Primorye 2024-1 (86.8)	Primorye 2024-3 (87.0)	0.96	1.57
Primorye 2024-2 (86.9)	Primorye 2024-3 (87.0)	0.93	2.47
Chita 2024-1 (87.4)	BT-1968, GenBank ID: LC790676 (86.9)	0.98	1.24
Khabarovsk 2024-1 (86.8)		0.97	1.61
Primorye 2024-1 (86.8)		0.96	1.66
Primorye 2024-2 (86.9)		0.93	2.33
Primorye 2024-3 (87.0)		0.99	0.88

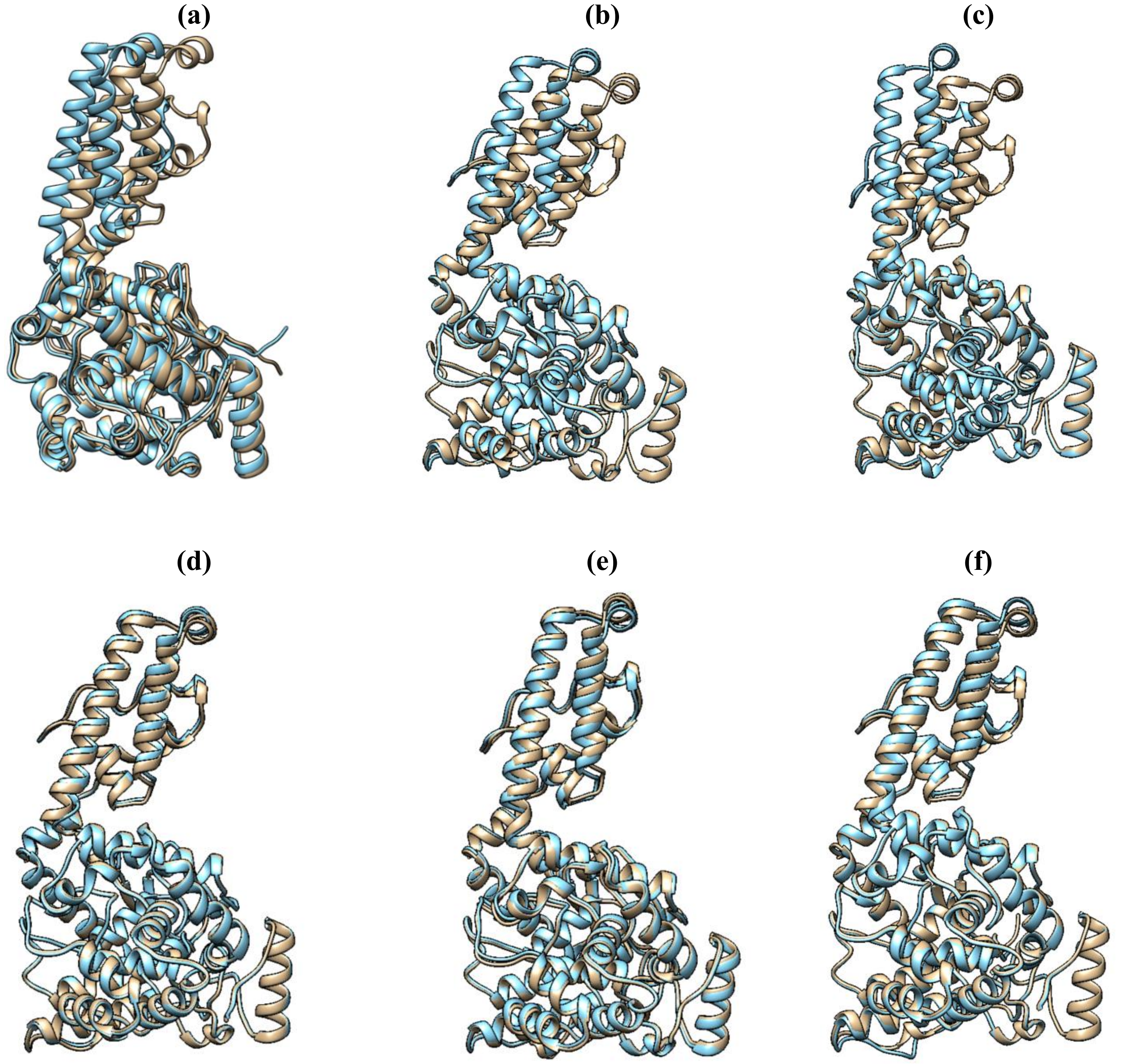

Figure S7. Imposition models of YEZV N tertiary structures. (**a**) Imposition models of YEZV N Chita 2024-1 isolate tertiary structure (ivory) with YEZV N Khabarovsk 2024-1 isolate (blue); (**b**) Imposition models of YEZV N Chita 2024-1 isolate tertiary structure (ivory) with YEZV N Primorye 2024-1 isolate (blue); **(c)** Imposition models of YEZV N Chita 2024-1 isolate tertiary structure (ivory) with YEZV N Primorye 2024-2 isolate (blue); **(d)** Imposition models of YEZV N Chita 2024-1 isolate tertiary structure (ivory) with YEZV N Primorye 2024-3 isolate (blue); **(e)** Imposition models of YEZV N Khabarovsk 2024-1 isolate tertiary structure (ivory) with YEZV N Primorye 2024-1 isolate (blue); (**f**) Imposition models of YEZV N Chita 2024-1 isolate tertiary structure (ivory) with YEZV N BT-1968 isolate (GenBank ID: LC790676, *I. persulcatus*, Japan) (blue).

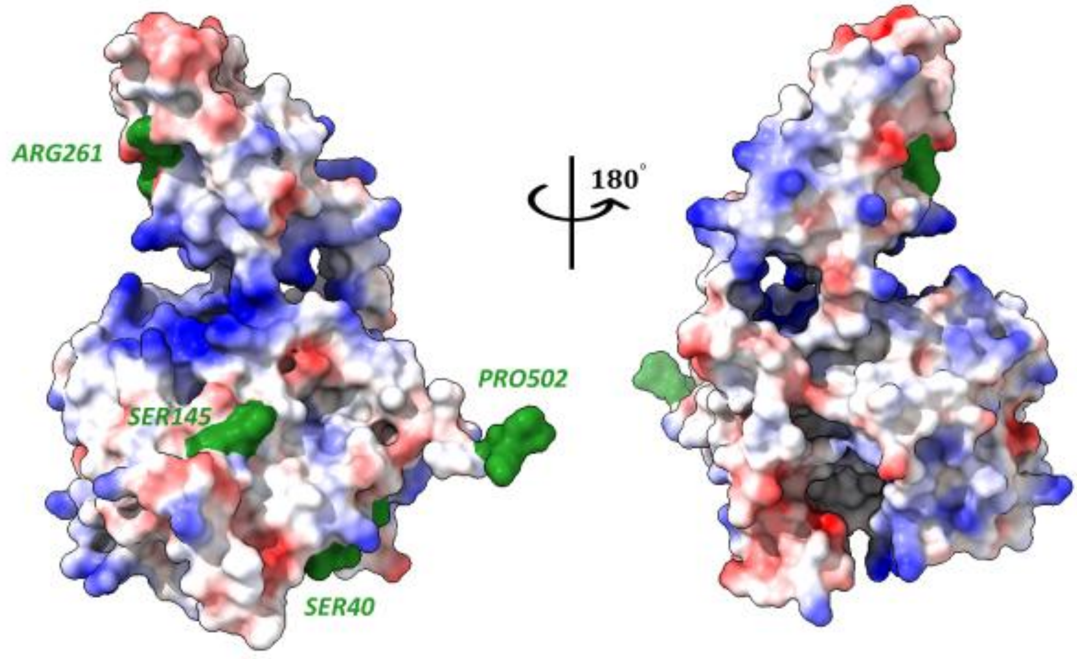

Figure S8. Electrostatic surface potential of YEZV N Chita 2024-1 isolate. The positive surface potential is colored blue, and the negative surface potential is colored red. YEZV RNA binding amino acids are highlighted in green.
